# A Transfer Learning Framework with a One-Dimensional Deep Subdomain Adaptation Network for Bearing Fault Diagnosis under Different Working Conditions

**DOI:** 10.3390/s22041624

**Published:** 2022-02-18

**Authors:** Ruixin Zhang, Yu Gu

**Affiliations:** 1 College of Information Science and Technology, Beijing University of Chemical Technology, Beijing 100029, China; rxzhang1@mail.buct.edu.cn; 2 Guangdong Province Key Laboratory of Petrochemical Equipment Fault Diagnosis, Maoming 525000, China; 3 Beijing Advanced Innovation Center for Soft Matter Science and Engineering, Beijing University of Chemical Technology, Beijing 100029, China; 4 Department of Chemistry, Institute of Inorganic and Analytical Chemistry, Goethe-University, Max-von-Laue-Str. 9, 60438 Frankfurt, Germany

**Keywords:** fault diagnosis, deep learning, rolling bearing, domain adaptation, transfer learning

## Abstract

Accurate and fast rolling bearing fault diagnosis is required for the normal operation of rotating machinery and equipment. Although deep learning methods have achieved excellent results for rolling bearing fault diagnosis, the performance of most methods declines sharply when the working conditions change. To address this issue, we propose a one-dimensional lightweight deep subdomain adaptation network (1D-LDSAN) for faster and more accurate rolling bearing fault diagnosis. The framework uses a one-dimensional lightweight convolutional neural network backbone for the rapid extraction of advanced features from raw vibration signals. The local maximum mean discrepancy (LMMD) is employed to match the probability distribution between the source domain and the target domain data, and a fully connected neural network is used to identify the fault classes. Bearing data from the Case Western Reserve University (CWRU) datasets were used to validate the performance of the proposed framework under different working conditions. The experimental results show that the classification accuracy for 12 tasks was higher for the 1D-LDSAN than for mainstream transfer learning methods. Moreover, the proposed framework provides satisfactory results when a small proportion of the unlabeled target domain data is used for training.

## 1. Introduction

Due to advances in industrial technology, rotating machinery is increasingly used in many fields, such as electric power generation, chemical production, and aerospace [1,2]. Rolling bearings are indispensable elements in rotating machines [3] and are the main source of faults in this equipment [4]. Rotating machines may operate under unfavorable conditions, such as high ambient temperatures, high humidity, and overload conditions, resulting in bearing malfunctions [5]. Bearing faults can cause significant damage to mechanical equipment [6]. Therefore, accurate and rapid methods for rolling bearing fault diagnosis are required to ensure the normal operation of rotating machinery.

In recent years, artificial intelligence methods, such as heuristic algorithm [7], expert knowledge-based methods [8], and deep learning (DL) models [9], have gained increasing attention in diverse fields. In particular, DL models have been broadly employed for machinery fault detection and diagnosis systems [10]. Most DL models, such as the long short-term memory network (LSTM) [11], deep belief network (DBN) [12], and convolutional neural network (CNN) [13,14,15], perform well if the datasets of the source domain and target domain tasks have the same distribution [16]. However, this assumption is rarely applicable in practical conditions. In many real-world applications, the working conditions during testing and training differ [17]. Therefore, the unlabeled testing data may not have the same distribution as the labeled training data, potentially leading to the misclassification of DL methods [18]. Thus, it is essential to consider the change in working conditions to improve the accuracy and efficiency of bearing fault diagnosis.

Transfer learning aims to extract information from one or more source tasks and apply it to a target task [19]. Deep domain adaptation (DDA), a branch of transfer learning, is designed to train a classifier or other predictor when the source domain data and target domain data have different distributions [20]. Since DDA can minimize the distribution discrepancy between different domains, it is well suited for solving cross-domain diagnosis tasks. Yang et al. [21] developed a bearing fault diagnosis framework based on a two-dimensional CNN and DDA. In this framework, multikernel maximum mean discrepancy (MK-MMD) was used for domain adaptation in four convolution layers. Although the method achieved an average accuracy value of 99.14% for 12 transfer learning tasks, the diagnostic accuracy was only 97.52% when substantial differences in working conditions existed. Wu et al. [22] converted raw data into two-dimensional time–frequency images using continuous wavelet transform (CWT) and proposed an accurate model consisting of a CNN and a deep adaptation network (DAN) for bearing fault diagnosis. The framework achieved a diagnostic accuracy score of more than 98% on the bearing fault dataset of Case Western Reserve University (CWRU). Although the method achieved satisfactory accuracy for transfer learning tasks, converting the vibration signal into images was computationally complex and time-consuming. Zhang et al. [23] proposed a domain adaptation framework using an adversarial learning strategy for machinery fault diagnostics. An instance-level weighted mechanism was also integrated to address the open-set problem. Jiao et al. [24] proposed a residual network to extract features from raw vibration data and combined the maximum mean discrepancy (MMD) with a domain adversarial strategy to align the domain distribution. The method obtained an average fault diagnosis accuracy value of 99.32% for 12 transfer learning tasks on the CWRU dataset. However, domain adversarial-based methods contain several loss functions and converge slowly. Although DDA approaches have been utilized for fault diagnosis, most methods (mapping-based and adversarial-based methods) assume that the global distribution differs for the target and source domains and try to reduce this difference. However, differences in the subdomain distribution of features and output labels among different working conditions are rarely considered. Unsatisfactory results could occur if the fine-grained information is not captured [25]. Therefore, a subdomain adaptation strategy that can exploit the local affinity to capture the fine-grained information of each category is incorporated to match the subdomain distributions of data from different working conditions.

In this paper, a novel one-dimensional lightweight deep subdomain adaptation network (1D-LDSAN) framework is proposed for bearing fault diagnosis under different working conditions. A 1D-CNN backbone is used to extract sufficient features from the raw data as input to a fully connected (FC) classifier, which diagnoses the faults accurately by utilizing advanced data features. A subdomain adaptation strategy is employed to match the subdomain distributions of data from different working conditions. The contributions of this paper are summarized as follows.

(1) A novel fault diagnosis framework (1D-LDSAN) consisting of a feature extraction module and a classification and adaptation module is proposed. The feature extraction module, a lightweight 1D-CNN backbone, is designed to extract a sufficient number of comprehensive and significant features of different faults from the raw vibration signal. The classification and adaptation module is used to classify the data and minimize the subdomain distribution discrepancy of the data from two domains to improve the classification performance.

(2) Comparative experiments are performed to verify the performance of the proposed framework on the CWRU dataset. Five other approaches, including deep domain confusion (DDC), a domain-adversarial neural network (DANN), a residual joint adaptation adversarial network (RJANN), a Wasserstein distance-guided multi-adversarial network (WDMAN), and a one-dimensional CNN, were evaluated for comparison to assess the performance of the 1D-LDSAN. The results demonstrate the effectiveness and superiority of the proposed framework.

The remaining parts of this paper are organized as follows. The theoretical background, the CNN, the domain adaptation, and MMD are introduced in Section 2. In Section 3, the details of the proposed 1D-LDSAN model are presented. The datasets, the experimental results, and the discussion are provided in Section 4. Finally, the conclusions are summarized in Section 5.

## 2. Related Works

### 2.1. Convolutional Neural Network

**Figure 1:**
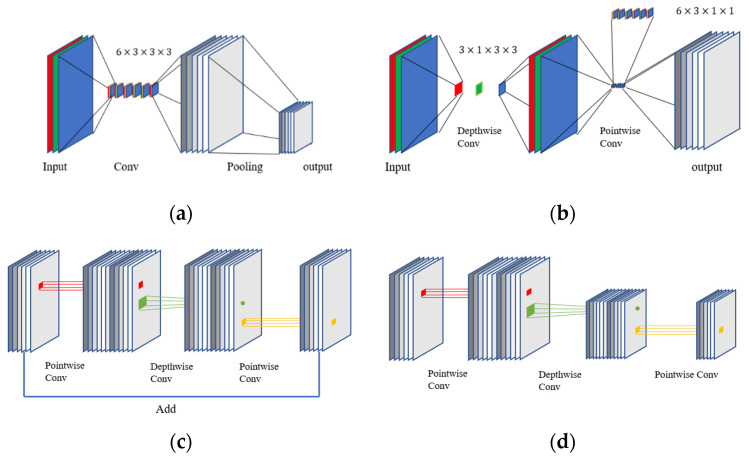
Comparison of convolutional blocks for different architectures. (**a**) Convolution pooling block; (**b**) separable block; (**c**) inverted residual block; (**d**) inverted residual block (stride).

A CNN is a multi-stage neural network composed of convolutional blocks and FC layers [26]. Traditionally, a convolutional block is composed of a convolution layer and a pooling layer [27], as shown in Figure 1a. In general, a batch normalization (BN) layer [28] is added after the convolution layer to improve the network training speed, prevent overfitting, and control gradient explosion and gradient disappearance. An activation function is required for nonlinear transformation after the convolution operation. The purpose of the activation function is to add nonlinear factors to the feature map after the convolution operation [29]. In this paper, the rectified linear unit (ReLU) [30] activation function is selected. Traditional CNNs require a pooling layer after activation to adjust the output of the convolution layer [31]. Many techniques have been used recently to replace the pooling function. MobileNet V2 [32] uses step convolution to replace the pooling layer. The classifier of a CNN is often an FC layer [33] that maps the learned features to the sample label space.

### 2.2. Domain Adaptation

Domain adaptation is a specific area of transfer learning; it refers to training a discriminative model in the presence of a domain shift between domains [34]. Domain adaptation establishes a knowledge transfer from the labeled source domain to the unlabeled target domain by using domain-invariant structures that bridge different domains with substantial discrepancies in the distribution [35,36].

In real-world applications, the working conditions of machines are often changed. Different working conditions are defined as different domains. The working condition with labeled data is defined as the source domain $D_{s}={\left\{x_{i},y_{i}\right\}}_{i=1}^{n}$, and the working condition with unlabeled data is defined as the target domain $D_{t}={\left\{x_{j}\right\}}_{j=1}^{m}$. It is assumed that they have the same feature space, $X_{s}=X_{t}$, and category space, $Y_{s}=Y_{t}$. However, the distributions of the two domains, $D_{s}$ and $D_{t}$, are different, $P_{s}\left(x_{s}\right)\neq P_{t}\left(x_{t}\right)$. The goal of this work is to use labeled source domain data $D_{s}$ and unlabeled target domain data $D_{t}$ to learn a classifier $f:x_{t}\to y_{t}$ to predict the labels $y_{t}\in Y_{t}$ of the target domain data $D_{t}$.

### 2.3. MobileNet V2

MobileNet V2 [32] is a lightweight CNN model for image processing. The main block in the model is inherited from the separable block [37], as shown in Figure 1b, and its main structure is combined with the residual structure [38] to construct an inverted residual block with a linear bottleneck. MobileNet V2 is created by embedding the inverted residual block instead of a standard convolution layer, as shown in Figure 1c,d. The linear bottleneck removes nonlinearities in the narrow layers because they destroy information in low-dimensional space. Hence, the linear bottleneck retains the representativeness of the model [39].

### 2.4. Local Maximum Mean Discrepancy (LMMD)

The MMD [40] calculates the discrepancy between two distributions by mapping sample points to the reproducing kernel Hilbert space (RKHS), which is a kernel method. Minimizing the MMD between the two domains aligns the edge probability distribution between the two domains in a neural network. The MMD between the source domain $D_{s}$ and target domain $D_{t}$ is defined as

(1) $$MMD^{2}\left(D_{s},D_{t}\right)=\|\frac{1}{n_{s}}\underset{x_{i}\in D_{s}}{\sum }\emptyset \left(x_{i}\right)-\frac{1}{n_{t}}\underset{x_{j}\in D_{t}}{\sum }\emptyset \left(x_{j}\right){\|}_{H}^{2}\;=\frac{1}{n_{s}^{2}}\overset{n_{s}}{\underset{i=1}{\sum }}\overset{n_{s}}{\underset{j=1}{\sum }}k\left(x_{i}^{s},x_{j}^{s}\right)+\frac{1}{n_{t}^{2}}\overset{n_{t}}{\underset{i=1}{\sum }}\overset{n_{t}}{\underset{j=1}{\sum }}k\left(x_{i}^{t},x_{j}^{t}\right)-\frac{2}{n_{s}n_{t}}\overset{n_{s}}{\underset{i=1}{\sum }}\overset{n_{t}}{\underset{j=1}{\sum }}k\left(x_{i}^{s},x_{j}^{t}\right)$$

where H represents the RKHS, ∅(·) denotes the feature map to map the raw sample to the RKHS, k is the kernel function $k\left(x^{s},x^{t}\right)=<\emptyset \left(x^{s}\right),\emptyset \left(x^{t}\right)>$, and <·,·> represents the inner product of two vectors.

The local MMD (LMMD) [25] is an improved version of the MMD for matching the local probability distribution. The LMMD between the source domain data $D_{s}$ and target domain data $D_{t}$ is calculated as follows:

(2) $$LMMD^{2}\left(D_{s},D_{t}\right)=\frac{1}{C}\overset{C}{\underset{c=1}{\sum }}\|\underset{x_{i}^{s}\in D_{s}}{\sum }\omega_{i}^{sc}\emptyset \left(x_{i}^{s}\right)-\underset{x_{j}^{t}\in D_{t}}{\sum }\omega_{j}^{tc}\emptyset \left(x_{j}^{t}\right){\|}_{H}^{2}\;=\frac{1}{C}\overset{C}{\underset{c=1}{\sum }}\left[\overset{n_{s}}{\underset{i=1}{\sum }}\overset{n_{s}}{\underset{j=1}{\sum }}\omega_{i}^{sc}\omega_{j}^{sc}k\left(x_{i}^{s},x_{j}^{s}\right)+\overset{n_{t}}{\underset{i=1}{\sum }}\overset{n_{t}}{\underset{j=1}{\sum }}\omega_{i}^{tc}\omega_{j}^{tc}k\left(x_{i}^{t},x_{j}^{t}\right)-2\overset{n_{s}}{\underset{i=1}{\sum }}\overset{n_{t}}{\underset{j=1}{\sum }}\omega_{i}^{sc}\omega_{j}^{tc}k\left(x_{i}^{s},x_{j}^{t}\right)\right]$$

where $x_{i}^{s}$ and $x_{j}^{t}$ represent the feature map of the source domain data and target domain data, respectively; $\omega_{i}^{sc}$ and $\omega_{j}^{tc}$ denote the weights of $x_{i}^{s}$ and $x_{i}^{t}$ belonging to class c, $\overset{n_{s}}{\underset{i=1}{\sum }}\omega_{i}^{sc}$ and $\overset{n_{t}}{\underset{j=1}{\sum }}\omega_{j}^{tc}$ are equal to 1, and $\underset{x_{i}^{\;}\in D_{\;}}{\sum }\omega_{i}^{c}\emptyset \left(x_{i}^{\;}\right)$ is the weighted sum of class C.

## 3. Materials and Methods

**Figure 2:**
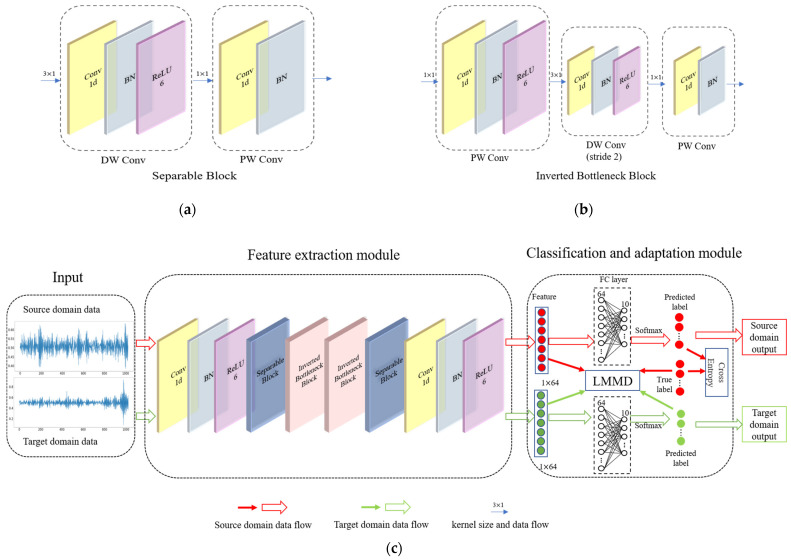
Schematic diagram of the proposed 1D-LDSAN framework. (**a**) is the structure diagram of the separable block shown in (**c**). (**b**) presents the structure diagram of the inverted bottleneck block shown in (**c**). (**c**) displays the overall framework.

Although DDA approaches have been used for fault diagnosis, most existing methods assume that the global distribution differs for the target and source domains and try to reduce this difference. However, differences in the subdomain distribution of features and output labels among different working conditions are rarely considered. Thus, fine-grained information in the categories may not be detected. Therefore, the 1D-LDSAN framework is proposed for the fault diagnosis of rolling bearings under different working conditions. As shown in Figure 2c, the proposed framework consists of a feature extraction module and a classification and adaptation module. The details will be introduced in the following subsections.

### 3.1. Framework Structure

#### 3.1.1. Feature Extraction Module

Inspired by MobileNet V2, we designed the feature extraction module to extract deep features from the raw data from the source domain and target domain. As shown in Figure 2c, the feature extraction module consists of two regular convolutional blocks and four unique convolutional blocks. The input size of the feature extraction module is 1024 × 1. The regular convolutional block consists of a convolutional layer, a BN layer, and a ReLU6 layer. The first regular convolutional block has a kernel size of 4 × 1 and a stride of 4 to reduce the length of input data. The second regular convolutional block has a kernel size of 1 × 1 and a stride of 1 to expand the number of feature channels. The unique convolutional blocks consist of two types: a separable block and an inverted bottleneck block, as shown in Figure 2a,b. The separable block is divided into two layers. The first layer is a depthwise convolution that performs lightweight filtering by applying a single convolutional filter per input channel. The second layer is a 1 × 1 convolution (pointwise convolution) responsible for creating new features by computing linear combinations of the input channels. The two-layer operation of the separable block replaces the full convolutional operator, which substantially reduces the number of parameters of the convolution kernel. In the inverted bottleneck block, a pointwise convolution is inserted in front of the separable convolution layer. In the second layer, the stride of the depthwise separable convolution is two. The structure maps the features to a high-dimensional space for fine-grained feature extraction. The details of the CNN backbone are listed in Table 1.

#### 3.1.2. Classification and Adaptation Module

As shown in Figure 2c, the classifier of the framework is an FC neural network. The weights of the classifier are shared by the source domain features and the target domain features. The number of neurons in the classifier is the same as the number of extracted features. The input of the LMMD function has four items, including the source domain features, the target domain features, the true label of the source domain data, and the predicted label of the target domain data. The classification and adaptation module is designed to minimize the classification errors of the source domain using a cross-entropy function and reduce the subdomain distribution discrepancy between the target domain and the source domain using the LMMD.

### 3.2. Optimization Objectives

This subsection describes the optimization objectives of the proposed framework. The framework has two optimization objectives, as shown in Figure 2c. The cross-entropy [41] function is implemented to minimize the classification error of the source domain dataset; it is defined as follows:

(3) $$L_{c}=-\overset{C}{\underset{c=1}{\sum }}y_{s}{}_{k}\times \log\frac{e^{{\tilde{y}}_{s}{}_{c}}}{\sum_{j}e^{{\tilde{y}}_{s}{}_{j}}}$$

where ${\tilde{y}}_{s}$ is the predicted label vector of the source domain data, $y_{s}$ is the true label vector of the source domain data, and C is the number of labels.

As described in Section 2.3, the LMMD is used to minimize the local subdomain distribution between the source domain data and target domain data. During training, the LMMD loss $L_{LMMD}$ is calculated as follows:

(4) $$L_{LMMD}=LMMD^{2}\left(x_{s},x_{t},y_{s},{\tilde{y}}_{t}\right)$$

where $x_{s}$ and $x_{t}$ are the features extracted from the source domain data and the target domain data, respectively. ${\tilde{y}}_{t}$ is the label vector of the target domain data predicted by the framework. $y_{s}$ is the true label vector of the source domain data.

The cross-entropy function and loss function are combined and represent the optimization goal, which is described as

(5) $$L=L_{c}+\lambda L_{LMMD}$$

where *λ* is the tradeoff parameter.

### 3.3. Network Training Strategy

Some DDA methods [18,42] must pre-train the neural network with the source domain data, which increases the training time and complicates the training process. This paper uses a strategy [20] of gradually increasing the tradeoff parameter λ from 0 to 0.2 during training, where $\lambda =0.2\times \left(2\;/\;\left(1+e^{\left(-10\times \left(epoch\right)\;/\;epochs\right)}\right)–1\right)$. An Adam [43] optimization strategy is used to optimize the network parameters, and a data augmentation algorithm is implemented to enhance network generalization. The data are jittered up and down randomly during training. Exponential attenuation of the learning rate is implemented to improve the stability of the framework during training. The learning rate has an initial value of 0.01 and decreases with an increase in the number of training epochs. The batch size is 64. During training, 80% of the source domain data and 50% of the unlabeled target domain data are used for domain-adaptive training. The remaining source data are used for validation, and the remaining target domain data are used for testing. The pipeline of training the 1D-LDSAN is presented in Algorithm 1.
**Algorithm 1** 1D-LDSAN.**Input:** labeled source domain data and unlabeled target domain data. **Output:** predicted category of target domain. **Begin** Step 1: normalize source domain and target domain data Step 2: initial neural network parameters with random values Step 3: input the normalized source domain and target domain data into the neural network to calculate $L_{c}$ and $L_{LMMD}$ Step 4: optimize the parameters of neural network using Adam strategy, repeat Step 3 and Step 4 until the specified epoch is reached Step 5: save the model Step 6: diagnose the target domain data using the trained model Step 7: output the classification results **End**

## 4. Experiments

In this study, the CWRU dataset [44] was used to evaluate the performance and practicability of the proposed 1D-LDSAN framework. Five other methods were evaluated for comparison. Pytorch 1.8.1 was used to implement the proposed framework, and a computer with the Windows 10 operating system and a gtx1050 GPU was used.

### 4.1. Dataset Description

**Figure 3:**
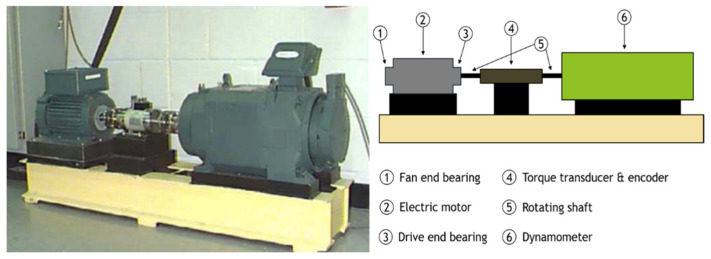
Photo (**left**) and schematic diagram (**right**) of the experimental device at Case Western Reserve University to assess bearing failure [44].

The CWRU dataset is a standard bearing fault dataset collected by the bearing center at CWRU. It is commonly used to validate and/or improve motor condition assessment techniques. Here, it was used to verify the performance of the framework. Figure 3 shows a photo and a diagram of the experimental platform. Bearings are used at the fan end and the drive end of the motor to enable the rotation of the motor’s shaft. The drive-end bearing is an SKF6205 deep groove ball bearing, and the fan-end bearing is an SKF6203 deep groove ball bearing. Two acceleration sensors were placed above the bearing pedestal at the fan end and drive end of the motor, respectively, to collect the vibration acceleration signal of the faulty bearing.

There are three fault types in this dataset, i.e., inner race fault (IF), outer race fault (OF), and roller fault (RF). The faults are machined by an electrical discharge machine (EDM), and each fault type has three damage sizes (0.007, 0.014, and 0.021 inches). Therefore, the CWRU dataset has ten classes (one normal class and nine fault classes (3 fault classes × 3 fault diameters)). The fault data were collected under four operating conditions in the experiment, including 0 HP, 1 HP, 2 HP, and 3 HP, with a sampling frequency of 12 kHz. Thus, the data were divided into four domains (A, B, C, and D), and there were 12 transfer learning tasks. The details of the dataset are presented in Table 2. Each sample contained 1024 data points.

### 4.2. Comparison of Different Signal Lengths

We extracted features from the raw bearing data using four signal lengths (256, 512, 1024, and 2048) to determine the optimum signal length for the 1D-LDSAN framework. Table 3 lists the number of samples obtained using the four signal lengths. In the experiment, the batch sizes were 256, 128, 64, and 32, respectively. A total of 12 transfer learning tasks were conducted. For example, transfer task A–B indicates that 0 HP is the source domain and 1 HP is the target domain.

The results are listed in Table 4. It was found that 1024 provided the best results and was used as the signal length. This experiment demonstrates the power of the proposed framework to model fault-related nonlinear vibration signals.

### 4.3. Comparison with Other Transfer Learning Methods

The detection accuracy of 1D-LDSAN was compared with that of five other methods, including a 1D-CNN, DDC [45], DANN [46], RJANN [24], and the WDMAN [47]. The 1D-CNN has the same architecture as the proposed 1D-LDSAN for feature extraction. It uses only the source domain samples to train a domain-shared 1D-CNN, and the model is tested with the target domain samples. The DDC is a mapping-based DDA method. DANN is a type of adversarial neural network. In this experiment, the 1D-CNN was employed as the feature extractor of the DDC and DANN. Each method was implemented with the optimal parameters. RJANN and WDMAN are other popular DDA methods used for fault diagnosis.

**Figure 4:**
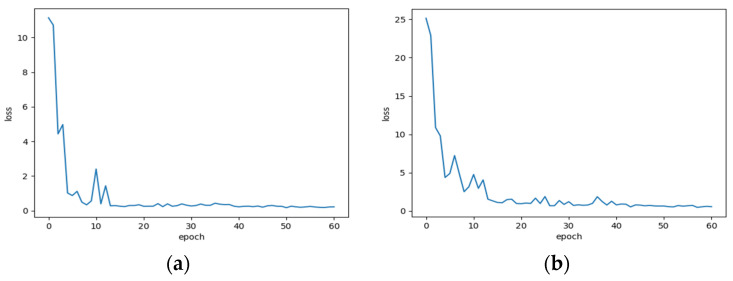
The validation loss of the 1D-LDSAN during the training on the CWRU dataset. (**a**) Source domain; (**b**) target domain.

Figure 4 displays the 1D-LDSAN’s validation loss during the training process. The convergence occurs after 50 epochs. Each experiment was repeated ten times for each model. The average detection results on the CWRU dataset are summarized in Table 5. The proposed 1D-LDSAN achieves an average detection accuracy score of 99.82%, outperforming the other five methods. Among the five comparison methods, the four deep transfer learning methods are superior to the DL method. Although the WDMAN and RJAAN achieve average classification accuracies above 99%, there are gaps between them and the proposed method for some transfer tasks. The 1D-CNN, DDC, and DANN achieve good results when the discrepancy is relatively small, such as the transfer task between A and B. However, the transfer task performance is unsatisfactory for the three methods when the working conditions change dramatically, resulting in low fault diagnosis accuracy. Notably, the proposed 1D-LDSAN exhibits excellent accuracy for all 12 transfer tasks. Therefore, these results demonstrate the effectiveness and superiority of the proposed method.

**Figure 5:**
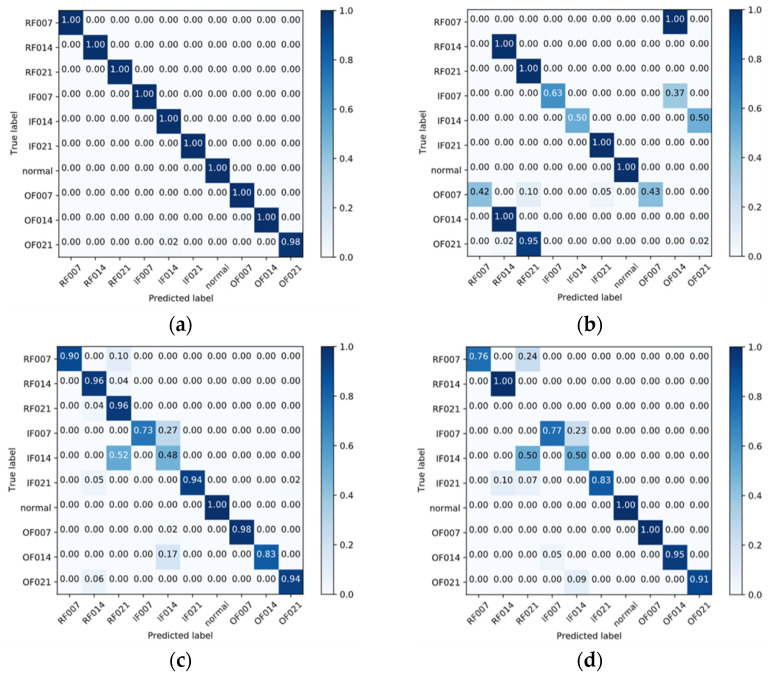
Confusion matrices of the different models for transfer learning task A–D (the color of the background becomes darker as the values get larger). (**a**) 1D-LDSAN; (**b**) 1D-CNN; (**c**) DDC; (**d**) DANN.

Figure 5 shows the confusion matrices of the 1D-LDSAN, 1D-LCNN, DDC, and DANN for the transfer learning task A-D. The proposed method achieves 100% accuracy for each condition, except for the label OF021. As shown in Figure 5b, the 1D-LCNN misclassifies many samples due to the significant distribution discrepancy between the domains. For example, almost all samples of OF014 are misclassified as RF014. In contrast, the DDC and DANN obtain better results than the 1D-LCNN.

**Figure 6:**
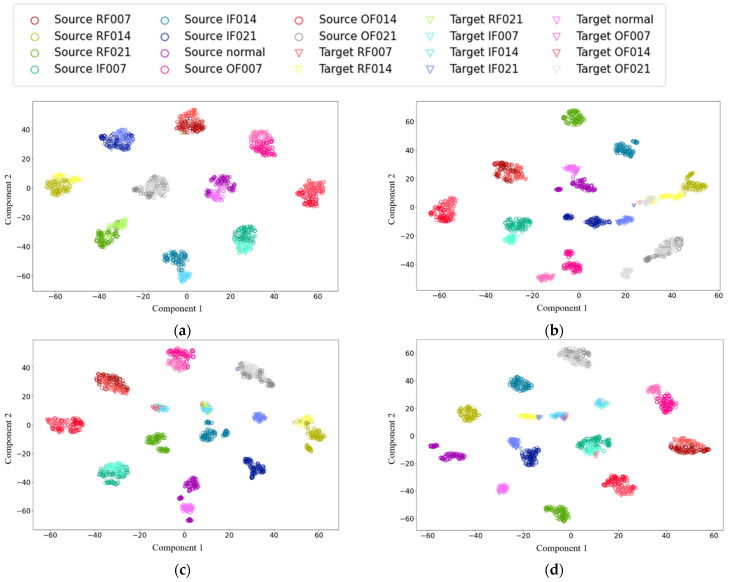
Visualization of features of the different models for the transfer learning task D-A using t-distributed stochastic neighbor embedding. (**a**) 1D-LDSAN; (**b**) 1D-CNN; (**c**) DDC; (**d**) DANN.

T-distributed stochastic neighbor embedding (t-SNE) [48] is used for nonlinear dimensionality reduction to visualize the features and analyze the domain adaptation and classification performance of the models. The visualization results of the 1D-LDSAN, 1D-LCNN, DDC, and DANN for the randomly chosen transfer learning task D-A are shown in Figure 6. It is observed that the proposed 1D-LDSAN produces more separated clusters (Figure 6a) than the 1D-CNN (Figure 6b) (no transfer learning). These results indicate that the 1D-LDSAN can better deal with the domain shift between the source and target domains. In contrast, there are substantial discrepancies between the source and target domains in the two domain adaptation methods (Figure 6c,d), resulting in many misclassifications. In summary, these results demonstrate that the proposed approach achieves more satisfactory classification performance and domain adaptation ability than the other methods.

### 4.4. Verification with a Small Proportion of the Target Domain Data

Different proportions of target domain data used for training produce different results. An experiment was conducted to determine the subdomain adaptation ability of the proposed model using a small proportion of the target domain data. We used six proportions of the target domain data for transfer learning (0%, 10%, 20%, 30%, 40%, and 50%, where 0% indicates no transfer learning). The remaining target domain data were used for testing.

As shown in Table 6, the classification accuracy improved from 0% to 10%, especially for tasks whose working conditions changed substantially. The accuracy of the 1D-LDSAN was high for the last five proportions, indicating that the proposed framework has good generalization performance for different percentages of the target domain data. Furthermore, when only 10% of the unlabeled target domain data were used for training in the 12 transfer tasks, the proposed model achieved more than 98% accuracy based on the remaining 90% of the target domain test data. The experimental results show that the proposed framework has strong feature extraction and domain adaptation ability and can extract sufficient information from a small proportion of the target domain data.

### 4.5. Parameter Sensitivity Analysis

**Figure 7:**
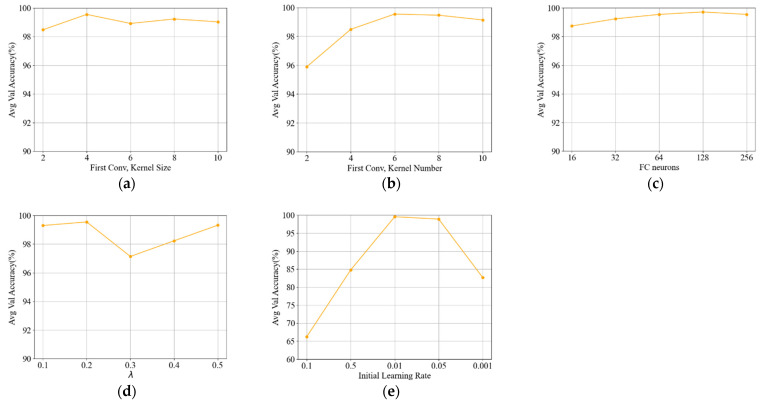
Effects of different parameters on the framework performance. (**a**) Kernel size of the first convolution layer; (**b**) kernel number of the first convolution layer; (**c**) neurons of the FC layer; (**d**) *λ*; (**e**) initial learning rate.

A sensitivity analysis of the five key parameters of the proposed framework was conducted. The results for the validation task are presented in Figure 7. The influence of multiple parameters was examined. It was found that the detailed framework architecture had a negligible influence on the model performance, except for the kernel number of the first convolution layer. The effects of the threshold parameter λ and the initial learning rate were also investigated. The results indicate that λ has a relatively small influence on the framework performance. It is worth noting that the initial learning rate has a marked influence on model performance.

## 5. Conclusions

We proposed the one-dimensional lightweight deep subdomain adaptation network (1D-LDSAN) to classify fault types of rolling bearings under different working conditions. The raw vibration signal was divided into small segments in the source and target domains. The advanced features in the segments were extracted by a one-dimensional lightweight convolutional neural network backbone. The local maximum mean discrepancy (LMMD) was employed to match the subdomain distributions, and the cross-entropy function was used to train a fully connected classifier using the labeled source domain data.

We compared the classification accuracy for different signal lengths and chose a length of 1024. The proposed 1D-LDSAN framework outperformed five other models for classifying rolling bearing faults on the CWRU dataset, indicating superior diagnosis performance. An experiment with six proportions of the target domain data for training indicated that the proposed framework could extract sufficient information from a small proportion of the target domain data, indicating excellent domain adaptation performance.

This study provides a solution for the intelligent fault diagnosis of rolling bearings and demonstrates the potential of domain adaptation for fault diagnosis under different working conditions. In a future study, we will focus on more effective deep domain adaptation methods.

## Figures and Tables

**Table 1:** Details of feature extraction module.

Block	Layer	Parameters	Output Size
Input	Input	/	1024 × 1
Regular Conv	ConvBNReLU6	Kernel size = 6@4 × 1 × 1 stride = 4	256 × 6
Separable Block	ConvBNReLU6	Kernel size = 6@3 × 1 stride = 1	256 × 6
	ConvBN	Kernel size = 16@1 × 1v4 stride = 1	256 × 16
Inverted Bottleneck Block	ConvBNReLU6	Kernel size = 96@1 × 1 × 16 stride = 1	256 × 96
	ConvBNReLU6	Kernel size = 96@3 × 1 stride = 2	128 × 96
	ConvBN	Kernel size = 24@1 × 1 × 96 stride = 1	128 × 24
Inverted Bottleneck Block	ConvBNReLU6	Kernel size = 144@1 × 1 × 24 stride = 1	128 × 144
	ConvBNReLU6	Kernel size = 144@3 × 1 stride = 2	64 × 144
	ConvBN	Kernel size = 32@1 × 1 × 144 stride = 1	64 × 32
Separable Block	ConvBNReLU6	Kernel size = 32@3 × 1 stride = 1	64 × 32
	ConvBN	Kernel size = 48@1 × 1 × 32 stride = 1	64 × 48
Regular Conv	ConvBNReLU6	Kernel size = 64@1 × 1 × 48 stride = 1	64 × 64
Avg Pooling	/	/	1 × 64

**Table 2:** Description of the CWRU dataset.

Domain	Load (HP)	Rotating Speed (r/min)	Number of Samples	Number of Labels
A	0	1797	1186	10
B	1	1772	1186	10
C	2	1750	1185	10
D	3	1730	1189	10

**Table 3:** Description of the samples of the four signal lengths.

Domain	256 Points	512 Points	1024 Points	2048 Points
A	4763	2379	1186	591
B	4762	2379	1186	591
C	4760	2377	1185	591
D	4769	2383	1189	592

**Table 4:** Classification accuracies of different signal lengths. The numbers in bold indicate the highest classification accuracy in corresponding task.

Task	256 Points	512 Points	1024 Points	2048 Points
A-B	98.84%	99.65%	99.90%	**99.93%**
A-C	97.04%	99.45%	99.89%	**99.90%**
A-D	97.44%	99.64%	**99.98%**	99.90%
B-A	98.94%	99.79%	**99.96%**	98.70%
B-C	99.11%	99.66%	**100.00%**	99.93%
B-D	95.96%	96.27%	**99.97%**	99.93%
C-A	97.95%	98.90%	**99.77%**	98.80%
C-B	98.44%	99.45%	99.55%	**99.73%**
C-D	98.70%	99.80%	99.93%	**99.97%**
D-A	94.16%	97.11%	**99.54%**	98.80%
D-B	92.42%	95.94%	**99.48%**	97.17%
D-C	98.28%	99.19%	**99.89%**	99.77%
AVG	97.27%	98.74%	**99.82%**	99.38%

**Table 5:** Classification accuracies of the different methods on the CWRU dataset. The numbers in bold indicate the highest classification accuracy in corresponding task.

Task	1D-CNN	DDC	DANN	WDMAN [15]	Task	1D-CNN
A-B	99.23%	98.12%	99.53%	99.73%	99.20%	**99.90%**
A-C	89.20%	93.61%	95.50%	99.67%	99.37%	**99.89%**
A-D	77.88%	84.36%	84.43%	**100.00%**	99.37%	99.98%
B-A	98.23%	98.34%	97.39%	99.13%	99.01%	**99.96%**
B-C	91.59%	98.47%	98.50%	**100.00%**	99.92%	**100.00%**
B-D	78.51%	79.41%	88.17%	99.93%	99.31%	**99.97%**
C-A	88.90%	88.94%	92.70%	98.53%	99.13%	**99.77%**
C-B	90.66%	92.57%	93.76%	**99.80%**	99.40%	99.55%
C-D	84.59%	90.03%	90.72%	**100.00%**	99.40%	99.93%
D-A	77.27%	78.69%	79.19%	98.07%	98.84%	**99.54%**
D-B	69.82%	72.33%	76.71%	98.27%	99.24%	**99.48%**
D-C	80.06%	83.61%	86.37%	99.53%	99.61%	**99.89%**
AVG	85.49%	88.21%	90.25%	99.39%	99.32%	**99.82%**

**Table 6:** Experimental results for different proportions of the target domain data. The numbers in bold indicate the highest classification accuracy in corresponding task.

Task	0%	10%	20%	30%	40%	50%
A-B	99.23%	99.79%	99.57%	99.84%	**99.92%**	99.90%
A-C	89.20%	99.90%	99.79%	99.90%	**99.94%**	99.89%
A-D	77.88%	98.79%	98.85%	99.58%	97.48%	**99.98%**
B-A	98.23%	99.90%	99.86%	99.76%	99.91%	**99.96%**
B-C	91.59%	99.78%	99.96%	99.98%	99.75%	**100.00%**
B-D	78.51%	99.94%	99.27%	99.10%	**99.99%**	99.97%
C-A	88.90%	99.47%	99.70%	99.71%	**99.87%**	99.77%
C-B	90.66%	99.52%	99.53%	99.57%	**99.65%**	99.55%
C-D	84.59%	99.48%	99.66%	99.78%	99.82%	**99.93%**
D-A	77.27%	99.20%	**99.61%**	98.30%	99.33%	99.54%
D-B	69.82%	98.09%	98.38%	99.30%	99.09%	**99.48%**
D-C	80.06%	99.68%	99.78%	99.53%	99.79%	**99.89%**
AVG	85.50%	99.46%	99.49%	99.53%	99.55%	**99.82%**

## Data Availability

Not applicable.
